# Estimating stroke-free and total life expectancy in the presence of non-ignorable missing values

**DOI:** 10.1111/j.1467-985X.2009.00610.x

**Published:** 2010-04

**Authors:** Ardo van den Hout, Fiona E Matthews

**Affiliations:** Medical Research Council Biostatistics UnitCambridge, UK

**Keywords:** Healthy life expectancy, Missing data, Multistate model, Selection model, Survival

## Abstract

A continuous time three-state model with time-dependent transition intensities is formulated to describe transitions between healthy and unhealthy states before death. By using time continuously, known death times can be taken into account. To deal with possible non-ignorable missing states, a selection model is proposed for the joint distribution of both the state and whether or not the state is observed. To estimate total life expectancy and its subdivision into life expectancy in health and ill health, the three-state model is extrapolated beyond the follow-up of the study. Estimation of life expectancies is illustrated by analysing data from a longitudinal study of aging where individuals are in a state of ill health if they have ever experienced a stroke. Results for the selection model are compared with results for a model where states are assumed to be missing at random and with results for a model that ignores missing states.

## 1 Introduction

Multistate models can be used to describe transitions between states over time. A three-state illness–death model describes a situation where the first two states are living states and the third state is the absorbing death state. If the first state is the healthy state and the second state is the illness state, healthy life expectancy (LE) at a specified time point (such as an age or a number of years after an operation) is the expected remaining lifetime spent in the first state.

It is important to know how total LE subdivides into LE in health and ill health. Survival is not only about total LE but also about whether or not remaining lifetime will be spent in good health. A statistical model where transitions between the states are described conditional on covariates values can help to investigate possible risk factors for the onset of ill health. An example of a longitudinal survey where the methodology can be applied is a study where patients are followed after a surgical operation. Other examples are studies where stages of a disease are monitored after an infection, or longitudinal epidemiological studies. The research in the current paper was suggested by an investigation into the occurrence of stroke, i.e. individuals are in state 2 if they have experienced one or more strokes.

The longitudinal data that will be analysed in this paper will be detailed later, but the design of the study is quite common. Individuals are followed up over time and their health status is measured at prescheduled interviews. The three-state illness–death model that describes the heath status is progressive, i.e. a transition from state 2 back to state 1 is not possible. Time between interviews can vary within and between individuals. Transition times for the death state are known exactly but times for the transitions from state 1 to state 2 are interval censored.

Often longitudinal studies will be subject to missing data. Regarding missing states, two forms of missing values can be defined: intermittently missing values and missing values due to dropout from the study. In the presence of right censoring at the end of follow-up, these two forms cannot always be distinguished. For example, if ‘·’ denotes a missing state, *c* denotes a right-censored state and the states are denoted by their number, the missing values in the sequence 1,1,·,·,*c* are of unknown origin if there is no additional information. In case the probability of observing a state depends on the state itself, missing states are called non-ignorable (Little and Rubin, 2002; Rubin, 1976). For example, if individuals do not undertake an interview because they are in state 2, then the missing states are non-ignorable.

This paper discusses a selection model that describes both the transitions between the three states and the probability of observing the living states. The fitted model is used to estimate healthy LE and total LE.

Cole *et al.* (2005) discussed a selection model for longitudinal data subject to non-ignorable missing values. Their selection model encompasses a multistate model and a missing data model. With regard to the model for the ignorable missing states, we use the same method. Our multistate model, however, is different. Cole *et al.* (2005) used a discrete time multistate model whereas we model time continuously, which enables us to take exact death times into account. In addition, our model allows for right censoring and time-dependent transition intensities. The latter are modelled by using age as a time-dependent covariate. Several reasearchers have considered the interval-censored continuous time multistate model, be it with or without exact death times and right censoring; see, for example, Jackson *et al.* (2003), Satten and Longini (1996), Kay (1986) and Kalbfleisch and Lawless (1985). Equally there are references on generic longitudinal data modelling with non-ignorable missing values; see, for example, Little (1995), Molenberghs *et al.* (1997) and Albert and Follman (2003). When there are no interval-censored transition times, there is an alternative way to specify and estimate multistate models; see Putter *et al.* (2007) and the references therein.

A continuous time multistate model that includes a model for non-ignorable missing values has not yet been formulated. Modelling of missing values is of interest especially in the field of healthy LE (Izmirlian *et al.*, 2000; Lièvre *et al.*, 2003) where the analysis of missing data is not yet fully developed.

Our model for the transitions between the states is a three-state model with a partial Markov property (Commenges (1999), page 319). The model cannot be called a Markov model as transition intensities are related to time-dependent covariates via log-linear regression. The model is also not semi-Markov since the time since entry to the state is unknown and not taken into account.

In this paper, intermittently missing values and missing due to dropout are not distinguished. Right censoring is modelled separately since it is a different process. We use logistic regression models for the probability that a state is observed. When intermittently missing values and missing due to dropout can be distinguished, there are three possibilities for the states: an observed state, an intermittently missing state and a state missing owing to dropout. For this situation, the same framework can be used, but the logistic regression models are to be replaced by three-category multinomial–logit regression models (see Li *et al.* (2007)). The model discussed can easily be extended to a three-state model where recovery from state 2 is possible. However, in that situation the partial Markov assumption is often less likely as a previous stay in state 2 might be of importance for the probability of a transition from state 1 to state 2.

There are several ways in which we can deal with missing values. In the application, we compare the results of our model with the results of two other approaches. The first approach consists of ignoring the missing states, and the second is a missingness at random (MAR) model (Rubin, 1976) treating missing states as intermittently censored without any further modelling of the censoring.

Section 2 introduces the model. In Section 3, we discuss the inference, including the estimation of LE. A simulation study in Section 4 shows that not taking into account non-ignorable missing states can lead to biased results. Section 5 discusses the application and Section 6 is the discussion.

## 2 The model

In the data, individuals are measured over time, where both time points and time intervals may vary between and within individuals. Let *t* denote the time since the start of the study. At time *t*≥0, the state of an individual is *X*_*t*_ ∈ {1,2,3}. States 1 and 2 are living states and state 3 is the absorbing death state. We assume that death if it occurred is always observed and that we have exact transition times for the death state. At *t*=0, *X*_*t*_≠3. Let *R*_*t*_ denote the observation indicator at time *t*, i.e. *R*_*t*_=1 if *X*_*t*_ is observed and *R*_*t*_=0 otherwise.

We shall model *X*_*t*_ and *R*_*t*_ jointly by using a selection model (Little and Rubin, 2002) and specifying a time continuous three-state model for the states and two logistic regression models for the observation indicator, i.e. one model for *R*_*t*_=1 conditional on *X*_*t*_=1, and one model for *R*_*t*_=1 conditional on *X*_*t*_=2.

We start with the three-state model. A transition from state *r* to state *s*, *r*≠*s*, occurs with intensity *q*_*rs*_(*t*), where *q*_*rs*_(*t*)≥0 for (*r*,*s*) ∈ {(1,2),(1,3),(2,3)}, and *q*_*rs*_(*t*)=0 for (*r*,*s*) ∈ {(2,1),(3,1),(3,2)}. The intensity *q*_*rs*_(*t*) represents the instantaneous risk of moving from state *r* to state *s* at time *t*. Intensities that are not restricted to 0 are regressed on covariate vector **z**(*t*) by the log-linear model 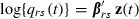, where ***β***_*rs*_=(*β*_*rs*.0_,.., *β*_*rs*.*p*_)^′^ and **z**(*t*)=(1,*z*_2_(*t*),.., *z*_*p*_(*t*))^′^.

We approximate the time dependence by assuming that the intensities do not change within an individually observed time interval. As a consequence, transition probabilities for a generic time interval (*t*_1_,*t*_2_] are given by the 3×3 matrix **P**(*t*_1_,*t*_2_)= exp {(*t*_2_−*t*_1_)**Q**(*t*_1_)}, where the transition intensity matrix **Q**(*t*) for time *t*≥0 is given by 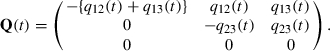
(1)

The *rs*-entry of **P**(*t*_1_,*t*_2_) is 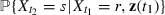, for *r*,*s* ∈ {1,2,3}. The piecewise constant approximation assumes a homogeneous time continuous three-state process within interval (*t*_1_,*t*_2_]. As a consequence, the above definition of **Q**(*t*) implies that **P**(*t*_1_,*t*_2_) is a stochastic matrix in the sense that every row is a distribution (Norris (1997), theorem 2.1.2).

In the application, time dependence of the intensities is modelled by using age as a time-dependent covariate. For an interval (*t*_1_,*t*_2_], age as a covariate is defined as age midway through the interval, i.e. as age at time (*t*_2_−*t*_1_)/2. This is possible because age is an external covariate in the sense that its value is known in advance at any future time (Collett (2003), section 8.1).

For the models for the observation indicator, we follow Cole *et al.* (2005) and define the conditional probability that *X*_*t*_=*x* is observed by 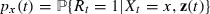, for *x* ∈ {1,2}. For ease of notation, we use the same covariate vector as above—other choices are available as will be illustrated in the application. We assume that the probabilities of observing the states can be described by logistic regression models 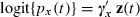, where ***γ***_*x*_=(*γ*_*x*.0_,…,*γ*_*x*.*p*_)^′^ for *x* ∈ {1,2}.

The selection model for the bivariate distribution of *X*_*t*_2__ and *R*_*t*_2__ for an observed time interval (*t*_1_,*t*_2_] is now given by 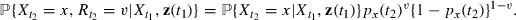
(2)

In what follows, we assume that the baseline state is always observed, i.e. *R*_0_=1, but modelling missing baseline states can be undertaken by extending the model.

## 3 Inference

### 3.1 Parameter estimation

Estimation of the parameters is undertaken by maximizing the log-likelihood. First, we describe the complete-data likelihood for a single individual with observation times *t*_1_,…,*t*_*M*_, where the state at *t*_*M*_ is allowed to be right censored. Let **x**^c^ denote the trajectory *x*_*t*_1__,…,*x*_*t*_*M*__. Owing to the partial Markov assumption, the contribution of the individual to the likelihood is given by 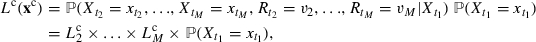
(3) where the conditioning of the covariates is suppressed in the notation. The contributions 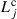 for *j* ∈ {2,…,*M*} are defined as follows. If the state observed at *t*_*j*_,*j* ∈ {2,..,*M*}, is 1 or 2, 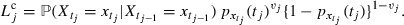


If the state *s* observed at *t*_*M*_ is death, 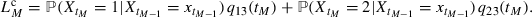


So we assume an unknown state at time *t*_*M*_ and then an instant death; see, for example, Satten and Longini (1996). If the state is right censored at *t*_*M*_, we assume that the individual is alive but with unknown state and define 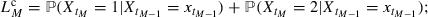 see, for example, Kay (1986).

Next missing states are taken into account and the likelihood contribution is derived by summing over all possible missing values, i.e. 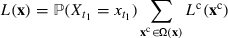 where Ω(**x**) is the set with all the trajectories where missing states are replaced by feasible latent states. Because our three-state model does not allow for recovery, only patterns with monotone increase are possible. For example, if **x**=*x*_*t*_1__,*x*_*t*_2__,*x*_*t*_3__,*x*_*t*_4__=1,·,·,2,3, then Ω(**x**)={(1,1,1,2,3),(1,1,2,2,3),(1,2,2,2,3)}.

Under the assumption that the parameters for the initial state and the subsequent transitions are distinct, we can ignore the initial state likelihood when estimating the multistate model (Cole *et al.*, 2005).

The formulation of the likelihood contributions resembles the formulation in Cole *et al.* (2005) where a discrete time model is specified. A continuous time model is defined by transition intensities, but, for maximum likelihood estimation, the intensities are translated to transition probabilities: hence the similarity in the likelihoods, even though the models are quite different. Taking into account exact death times and right censoring is an extension of the model in Cole *et al.* (2005).

Given the above three-state model without recovery where death is monitored, a missing state after a previously observed state 2 is always state 2. For example, if **x**=*x*_*t*_1__,*x*_*t*_2__,*x*_*t*_3__,*x*_*t*_4__=1,2,·,3, then we know that *x*_*t*_3__=2. The fact that state *x*_3_ is not observed is used in the estimation of the logistic regression model for the probability of observing state 2. Because we do not want to lose information on the missing data mechanism, we do not impute these kinds of missing state before the data have been analysed even though we know the unobserved states. In the application, we briefly discuss the effect of this approach.

### 3.2 Life expectancies

We assume that time-dependent covariate vector **z**(*t*) is external in the sense that **z**(*t*) is known for *t*>0, given **z**(0). Expected LE in state *s* ∈ {1,2} given initial state *r* ∈ {1,2} and 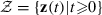 is given by 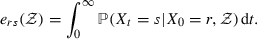
(4)

To estimate LE in state *s* irrespectively of the initial state, we need a model for the baseline state. Let 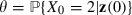. We use the logistic regression model logit(*θ*)=***α***^′^ **z**(0), where ***α***=(*α*_0_,…,*α*_*p*_)^′^. Marginal LE *e*_*s*_ and total LE *e* are now given by 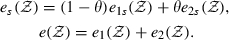


In the estimation of the three-state model, intensities were assumed to be constant within an individually observed time interval. To estimate integral (4), we use an analogous piecewise constant approximation. Firstly, we specify covariate values **z**(0) at baseline and create a time grid *u*_1_=0,*u*_2_,…,*u*_*M*_, where the time between two time points, say *h*, is fixed. Secondly, for each time interval (*u*_*j*_,*u*_*j*+1_] we specify **z**(*u*_*j*_). Finally, we numerically approximate integral (4) by using the trapezoidal rule with grid *u*_1_,*u*_2_,…,*u*_*M*_. To compute the integrand at a given grid point *u*_*j*_,*j*=2,…,*M*, we use the multiplication **P**(*u*_1_,*u*_2_) **P**(*u*_2_,*u*_3_)×…×**P**(*u*_*j*−1_,*u*_*j*_) to approximate **P**(*u*_1_,*u*_*j*_).

Given the recursive scheme above, it is not straightforward to compute the variance of LEs by the delta method, but it is easy to estimate the variance by simulation, i.e. we consider the multivariate normal distribution with expectation equal to the maximum likelihood estimate (MLE) of the parameter vector and the covariance matrix equal to the estimated covariance matrix at the optimum. By drawing parameter values from this distribution and computing the LEs for each of the values drawn, the sample variation in the estimation of the LEs will be reflected (see Aalen *et al.* (1997)). An alternative is to apply a Metropolis algorithm to capture the variation around the maximum of the log-likelihood. This is in the spirit of Tanner who pointed out the use of the algorithm in estimating some functional of the likelihood (Tanner, 1996). Yet another method, which was used in Aalen *et al.* (1997), is to apply the bootstrap by resampling from the data.

This simulation with maximum likelihood is fast and easy to apply but relies on asymptotic properties of maximum likelihood. The advantage of the Metropolis algorithm is that the estimation does not rely on asymptotic properties. Especially if the sample size is small, this can be important. The disadvantage of the Metropolis algorithm is that it is computationally intensive. The bootstrap is also computationally intensive because the model must be estimated in every iteration. The advantage of the bootstrap, however, is that it includes model uncertainty in addition to sampling uncertainty (Aalen *et al.*, 1997). In the application, we compare the maximum likelihood simulation with the Metropolis algorithm. The bootstrap was not feasible owing to computational limitations.

## 4 Simulation study

Even though it seems reasonable to take into account missing states, it is not directly evident that this will lead to better data analysis. A small simulation study will be conducted to investigate the performance of the selection model in comparison with two other three-state models. The two alternative models can handle missing states but do not take non-ignorable missingness into account. Denote the selection model in the previous section model 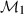. Next we define model 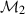 which explicitly allows for missing states but does not model a missing data mechanism, and model 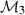 that ‘handles’ missing states by simply ignoring them. To illustrate 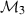, if an observed series of states is given by *x*_*t*_1__,*x*_*t*_2__,*x*_*t*_3__=1,·,3, then only the complete data given by *x*_*t*_1__,*x*_*t*_3__=1,3 are analysed.

For model 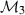, we formulate the complete-data likelihood for an individual with observation times *t*_1_,…,*t*_*M*_. The contribution of the individual to the likelihood is given by 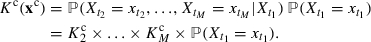


If the state that is observed at *t*_*j*_, *j* ∈ {2,…,*M*}, is 1 or 2, 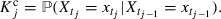


If the state *s* that is observed at *t*_*M*_ is death, 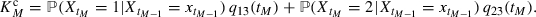


If the state is right censored at *t*_*M*_, 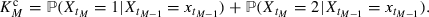


For model 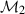, the same formulae as in 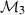 are used but, owing to missing states, **x**^c^ is not always observed. The full likelihood contribution of an individual is derived by summing over all possible missing values, i.e. 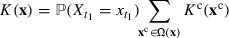 where Ω(**x**) is the set with all the trajectories where missing states are replaced by feasible latent states.

Models 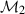 and 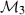 can be found in the literature on multistate models as referred to in Section 1. The models can be estimated by using the package in R (Jackson *et al.*, 2003). If there are missing states in the data, model 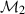 can be fitted to the data immediately. Note that 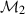 is an MAR model. For example, if **x**=*x*_*t*_1__,*x*_*t*_2__,*x*_*t*_3__=1,·, 3, then Ω(**x**)={(1,1,3),(1,2,3)}. Which element of Ω(**x**) is most likely depends on *x*_*t*_1__ and *x*_*t*_3__, but not on the unobserved *x*_*t*_2__. Data for model 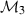 can be derived from the observed data by simply deleting all the intermittently missing states.

Van den Hout and Matthews (2009) show how data can be simulated given a time continuous multistate model with age as time-dependent covariate. In the present simulation study, we used a balanced design regarding age, i.e., given sample size *n*=200, we have age at baseline given by the integers 60, 61, …, 89, where each integer corresponds to the age of eight individuals. We simulate a follow-up of 14 years with observations every 2 years. At baseline, 10% of the individuals are in state 2. States of individual *i* are observed at times *t*_*ij*_ for *j*=1,…,*M*_*i*_, where *t*_*i*1_=0, and *M*_*i*_−1 is the number of planned interviews for individual *i*, and the state at *t*_*M*_*i*__ is either censored or the death state. Data are simulated by using 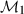. Given an interval (*t*_*ij*_,*t*_*i*,*j*+1_], the model for the intensities and the probability of an observed state are given by 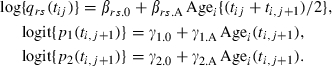
(5)

Age in the models is centred by subtracting 78.5 years and the covariate is evaluated midway through the interval. For the parameters values in equation (5) we choose (*β*_12.0_,*β*_13.0_,*β*_23.0_) equal to (−6.4,−5.4,−4.5), and (*β*_12.A_,*β*_13.A_,*β*_23.A_) equal to (0.10,0.06,0.05). The study design and the parameter values for the three-state model reflect the situation in the application. For the missing data model, we choose (*γ*_1.0_,*γ*_2.0_)=(2.0,0.5) and (*γ*_1.A_,*γ*_2.A_)=(−0.1,−0.2), which correspond to more missing values for state 2 and an increase of missing values for the older ages. These parameter values reflect a higher likelihood of missing states than the estimated parameter values in the application. Owing to computational limitations our simulation study is small with respect to sample size, and (*γ*_1.0_,*γ*_2.0_) and (*γ*_1.A_,*γ*_2.A_) are chosen such that they illustrate the difference between the models of interest. After simulating the data by using the specified parameter values and by imposing the study design, models 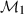, 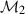 and 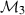 are used to estimate the parameters. For models 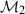 and 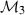 the equation for the intensities is given by equation (5).

Table 1 shows the results of the simulation study. Looking at the bias and the actual coverage percentage (ACP) of estimated 95% confidence intervals, model 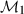 seems to have a better overall performance than the other two models. An exception is the estimation of *β*_13.A_, where model 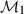 induces a larger bias. The ACPs for model 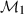 are slightly less than their nominal value. This may be caused by the sample size that is too small to fulfil the requirements of the asymptotic properties with respect to the maximum likelihood estimation. The performance of models 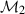 and 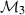 seems similar. These models induce severe bias in the estimation of *β*_12.0_, *β*_13.0_ and *β*_12.A_. The models underestimate the risk of moving from state 1 to state 2 and overestimate the risk of moving from state 1 to state 3—a bias that is absent for model 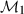.

**Table 1:** Results for the simulation study with sample size *n*=200 and 400 replications†

*Parameter*	*True value*	*Results for model* 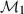		*Results for model* 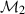		*Results for model* 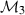	
		*Bias*	*ACP*	*Bias*	*ACP*	*Bias*	*ACP*
*β*_12.0_	−6.40	−0.040	0.95	−0.485	0.72	−0.422	0.78
*β*_13.0_	−5.40	0.034	0.92	0.075	0.86	0.102	0.83
*β*_23.0_	−4.50	0.065	0.93	0.036	0.95	0.080	0.93
*β*_12.A_	0.10	0.005	0.92	−0.065	0.61	−0.067	0.58
*β*_13.A_	0.06	−0.012	0.90	0.001	0.95	0.001	0.95
*β*_23.A_	0.05	−0.005	0.93	−0.003	0.93	0.004	0.97
*γ*_1.0_	2.00	0.105	0.92				
*γ*_2.0_	0.50	0.211	0.91				
*γ*_1.A_	−0.10	0.002	0.94				
*γ*_2.A_	−0.20	−0.010	0.94				

† Bias is the mean of the bias of the parameters estimates.

Even though the simulation study is limited in scope, it is clear that the presence of non-ignorable missing states can lead to biased inference when the missing data mechanism is ignored in the multistate analysis.

## 5 Application

First we briefly discuss the Medical Research Council ‘Cognitive function and ageing study’ (CFAS) (Brayne *et al.*, 2006). Next the three-state illness–death model will be defined where individuals are in state 2 when they have experienced one or more strokes and in state 1 if they have never had a stroke. This model was discussed in the previous sections. It will be denoted by 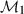 and it includes the modelling of non-ignorable missing states. We shall estimate healthy LE, which is defined as the remaining years of life spent stroke free.

As in Section 4, model 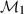 will be compared with two other models: 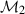 is an MAR model and for 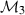 we ignore all intermittently missing states and estimate a complete-data model from the observed states only.

### 5.1 Medical Research Council ‘Cognitive function and ageing study’

The Medical Research Council CFAS is a population-based longitudinal study of cognition and health in the older population of England and Wales. Interviews were conducted in five centres in England and Wales: Oxford, rural Cambridgeshire, Nottingham, Gwynedd and Newcastle. The study was designed to have 2500 individuals in each centre. There were 13 004 individuals who undertook the baseline interview in 1991 and since then they have had up to eight interviews in the period 1991–2004. All individuals were aged 65 years and above at baseline, and all deaths up to the end of 2005 have been included. The time between interviews varies between and within individuals and the number of interviews is not fixed. The last observed state of an individual at the end of December 2005 is either death or censored and does not count as an interview.

Of interest are LE free from stroke and total LE. In what follows we describe and analyse the data from the Newcastle centre with 2512 individuals. At baseline there are 187 individuals with severe cognitive impairment. For this group, information on stroke history is missing or potentially unreliable. Although missing states at baseline can be incorporated in our model, the reliability of observed states poses problems that fall outside the scope of this paper. We therefore restrict our analysis to individuals who are not cognitively impaired at baseline. This also make sense clinically. Stroke is a potential cause of cognitive impairment and hence the stroke prevalence may not be estimated well for the cognitively impaired. Of the remaining 2325 individuals, four have a missing state at baseline. We removed these individuals from the data as modelling missing the baseline state for such a small group is not worthwhile. Hence there are 2321 individuals (880 men; 1441 women) in the analysis.

A state is defined to be missing if there is no interview at the scheduled time or if there is an interview but there is no information about stroke. The total number of interviews in which a state 1 or state 2 was observed is 5704. The total number of missing states is 2115, and the total number of censored states is 766. During follow-up 1555 individuals died. The mean length of time between interviews where a state 1 or state 2 was observed is 22.91 months (standard deviation 21.35). The mean length of time between dates with either a living state, the death state, a missing state or a censored state is 31.02 months (standard deviation 27.46).

**Fig. 1:**
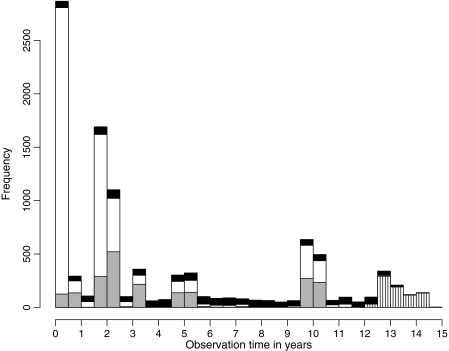
Observation times measured in years since baseline and corresponding states: ▪, death state; □, state 1 or 2; 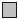, missing state; 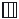, censored state

Fig. 1 provides information on the observation times for the four states. The CFAS had planned follow-up for all individuals at years 0, 2 and 10. Additional interviews were undertaken on selected individuals at years 1, 3, 6 and 8. These individuals are identified at previous interviews and hence missing interviews are easily identified.

Table 2 presents frequencies of observed transitions between the states in the CFAS when missing states are included and when missing states are removed. Note that there are no transitions from state 2 to state 1. There are quite a large number of missing states and Table 2 shows that there are also many missing states that are followed by a missing state, i.e. there are 1200 transitions from a missing state to a missing state. In the CFAS, individuals who refused one interview are not allowed to be recontacted owing to ethical constraints. The figures in the second part of Table 2 can partly be derived from the first part: 2966=24+2942 and 113=8+105. It is, however, only given the second part that it becomes clear that 224−176=48 out of the 542 transitions from a missing state to state 3 are due to missing states in between an observed state 2 and observed death. Consequently, 542−48=494 transitions from a missing state to the death states are due to individuals with one or more missing states between an observed state 1 and observed death.

**Table 2:** Frequencies of observed transitions between the states in the CFAS when missing states are included and when missing states are ignored

		*Frequencies of transitions to the following states:*				
		*Censored*	*Missing*	*1*	*2*	*3*
*Including missing states*						
From state	Missing	341	1200	24	8	542
	1	382	855	2942	105	837
	2	43	60	0	304	176
*With missing states ignored*						
From state	1	711		2966	113	1331
	2	55		0	304	224

In general, a missing state is not a complication for a continuous time model. The model allows for varying times between interviews. If an individual misses an interview for unrelated reasons, then that is not a violation of model assumptions. However, if an individual misses an interview because he or she recently had a stroke, then the missing state is non-ignorable and if we ignore this scenario we could bias the estimates. Another problem with ignoring missing states is that the approximation to the time dependence of the intensities becomes more coarse. For example, if the sequence is given by *x*_*t*_1__,*x*_*t*_2__,*x*_*t*_3__,*x*_*t*_4__=1,·,·,3 and the missing states are ignored, then the time dependence of the intensities in (*x*_*t*_1__,*x*_*t*_4__] is approximated by using age at (*x*_*t*_1__+*x*_*t*_4__)/2, whereas, if the missing states are not ignored, the time dependence is approximated by using age at (*x*_*t*_1__+*x*_*t*_2__)/2,(*x*_*t*_2__+*x*_*t*_3__)/2 and (*x*_*t*_3__+*x*_*t*_4__)/2.

### 5.2 Analysis

Time in the three-state model is the time since baseline in months. States of individual *i* are observed at times *t*_*ij*_ for *j*=1,…,*M*_*i*_, where *t*_*i*1_=0, and *M*_*i*_−1 is the number of planned interviews for individual *i*, and the state at *t*_*M*_*i*__ is either censored or the death state. The model for the baseline state is given by 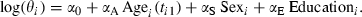
(6)

To model possible time-dependent transition intensities, age in years is added as a time-dependent covariate in the log-linear model for the intensities. For interval (*t*_*ij*_,*t*_*i*,*j*+1_], *j* ∈ {1,…,*M*_*i*_−1}, the model for the intensities and the probability of an observed state are given by 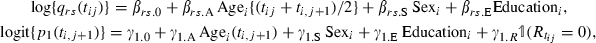
(7)
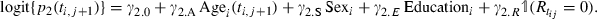
(8)

Age in the models is centred by subtracting 78.5 years. Sex and Education are dummy variables (men ≡ 1, more than 9 years of full-time education ≡ 1). Note that age as a covariate for the piecewise constant intensities is evaluated midway through the interval.

The selection model 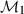 that includes the three-state model and the model for the missing states is estimated by maximizing the log-likelihood in the programming environment R (R Development Core Team, 2008) by using the general purpose optimizer. Within we choose the quasi-Newton Broyden–Fletcher–Goldfarb–Shanno method. The estimated Hessian is used to derive standard errors. The logistic regression model is estimated in R by using the function for generalized linear models. Parameters for the logistic regression model and the parameters for the three-state model are assumed to be distinct and the models are estimated separately.

Model 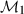 has −2 × log-likelihood = 23975.92, whereas the same model with the restriction *γ*_1.*R*_=*γ*_2.*R*_=0 has −2 × log-likelihood = 26252.87. This difference in the log-likelihood shows that the model with unrestricted *γ*_1.*R*_ and *γ*_2.*R*_ is better and that whether or not the previous state was observed helps to estimate the probability of observing the current state.

Table 3 shows the parameter estimates. As expected, the effect of age on the transition intensities is positive (*β*_12.A_,*β*_13.A_,*β*_23.A_>0) and men have an increased risk of a transition to stroke as well as to mortality (*β*_12.S_,*β*_13.S_,*β*_23.S_>0). Estimated models (7) and (8) for the probabilities of observing a state show that the older you are the more likely it is that your state is not observed and that this age effect is stronger for state 2 than for state 1. Estimated model (6) for the baseline state shows that the probability of having a stroke increases with age (*α*_A_>0).

**Table 3:** Parameter estimates (with standard errors in parentheses)

		*Results for model* 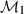		*Results for model* 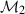		*Results for model* 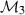	
*Three-state model*							
(Intercept)	*β*_12.0_	−6.441	(0.152)	−6.454	(0.144)	−6.294	(0.146)
	*β*_13.0_	−5.401	(0.055)	−5.403	(0.052)	−5.295	(0.052)
	*β*_23.0_	−4.534	(0.101)	−4.539	(0.103)	−4.420	(0.097)
Age	*β*_12.A_	0.103	(0.016)	0.078	(0.018)	0.065	(0.018)
	*β*_13.A_	0.062	(0.007)	0.070	(0.006)	0.065	(0.006)
	*β*_23.A_	0.050	(0.009)	0.046	(0.010)	0.039	(0.010)
Sex	*β*_12.S_	0.272	(0.193)	0.377	(0.195)	0.274	(0.200)
	*β*_13.S_	0.308	(0.080)	0.296	(0.075)	0.266	(0.075)
	*β*_23.S_	0.388	(0.122)	0.396	(0.127)	0.332	(0.126)
Education	*β*_12.E_	0.345	(0.225)	−0.045	(0.228)	−0.168	(0.236)
	*β*_13.E_	−0.395	(0.124)	−0.256	(0.093)	−0.254	(0.090)
	*β*_23.E_	0.180	(0.137)	0.123	(0.154)	0.067	(0.155)
*Models for probability of an observed state*				*Model for baseline state*			
(Intercept)	*γ*_1.0_	1.097	(0.056)	(Intercept)	*α*_0_	−2.475	(0.116)
	*γ*_2.0_	1.305	(0.184)	Age	*α*_A_	0.036	(0.012)
Age	*γ*_1.A_	−0.014	(0.007)	Sex	*α*_S_	0.369	(0.165)
	*γ*_2.A_	−0.066	(0.020)	Education	*α*_E_	−0.571	(0.213)
Sex	*γ*_1.S_	0.288	(0.089)				
	*γ*_2.S_	0.569	(0.286)				
Education	*γ*_1.E_	0.430	(0.108)				
	*γ*_2.E_	−0.533	(0.316)				
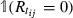	*γ*_1.R_	−5.057	(0.211)				
	*γ*_2.R_	−4.180	(0.394)				

**Fig. 2:**
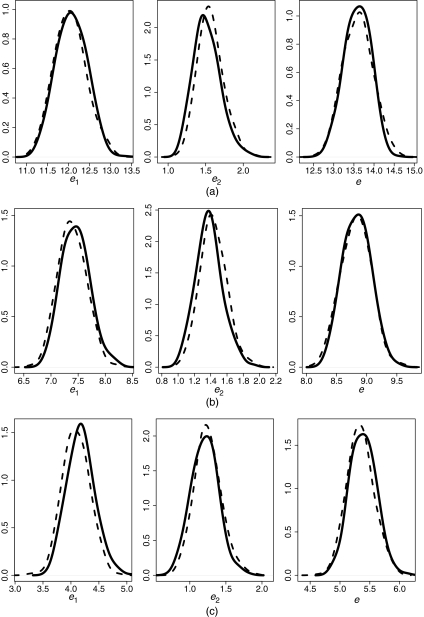
Estimated densities of LEs *e*_1_, *e*_2_ and *e* for men aged (a) 65 years, (b) 75 years and (c) 85 years at baseline with mean education level: a comparison between MLE simulation (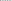) and the Metropolis algorithm (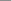)

To estimate LEs we use the piecewise constant approximation as explained in Section 3. Grid *u*_1_,*u*_2_,…,*u*_*M*_ for the trapezoidal rule is defined by using *h*=1 month. In what follows, the conditioning of the LEs on 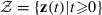 is suppressed in the notation. Table 4 reports point estimates and standard errors that are derived from simulating model parameter uncertainty using the MLE (500 simulated parameter vectors). The mean of the point estimates in the simulation can differ from point estimates that are calculated by using the MLE directly. This is due to possible skewness of the density of the LEs—even though the model parameters are simulated from a multivariate normal distribution. As an example, for men aged 65 years at baseline, *e*_22_ is estimated to be 7.15 years by using the mean of the MLE simulations and 7.08 years by using the maximum likelihood point estimate for the model parameters in equation (4). It is only for *e*_22_ that the figures differ slightly—the densities of the other LEs are close to symmetrical; see Fig. 2 for some of these densities.

**Table 4:** Estimated LEs (with estimated standard errors in parentheses) for women and men given mean level of education

*Parameter*	*Results for model* 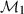		*Results for model* 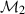 , *men*	*Results for model* 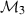 , *men*
	*Women*	*Men*		
*Aged 65 years at baseline*				
*e*_11_	15.11 (0.36)	12.82 (0.37)	12.72 (0.26)	12.04 (0.25)
*e*_12_	1.66 (0.19)	1.20 (0.15)	1.28 (0.12)	1.25 (0.11)
*e*_22_	9.55 (0.85)	7.15 (0.69)	7.06 (0.52)	6.50 (0.49)
*e*_1_	14.46 (0.36)	12.04 (0.37)	11.93 (0.27)	11.30 (0.27)
*e*_2_	2.00 (0.19)	1.57 (0.17)	1.64 (0.13)	1.58 (0.13)
*e*	16.46 (0.35)	13.60 (0.36)	13.57 (0.24)	12.87 (0.23)
*Aged 75 years at baseline*				
*e*_11_	9.77 (0.26)	8.07 (0.26)	8.08 (0.18)	7.85 (0.18)
*e*_12_	1.57 (0.20)	1.12 (0.16)	1.05 (0.10)	1.02 (0.10)
*e*_22_	6.55 (0.41)	4.78 (0.36)	4.85 (0.28)	4.70 (0.27)
*e*_1_	9.18 (0.25)	7.39 (0.25)	7.38 (0.19)	7.18 (0.19)
*e*_2_	1.87 (0.20)	1.43 (0.16)	1.38 (0.11)	1.33 (0.11)
*e*	11.05 (0.23)	8.82 (0.25)	8.76 (0.16)	8.51 (0.17)
*Aged 85 years at baseline*				
*e*_11_	5.76 (0.28)	4.64 (0.25)	4.77 (0.18)	4.84 (0.18)
*e*_12_	1.42 (0.24)	1.00 (0.19)	0.82 (0.12)	0.79 (0.12)
*e*_22_	4.36 (0.33)	3.12 (0.30)	3.26 (0.25)	3.36 (0.28)
*e*_1_	5.27 (0.27)	4.09 (0.24)	4.21 (0.18)	4.27 (0.19)
*e*_2_	1.67 (0.23)	1.25 (0.18)	1.11 (0.13)	1.10 (0.13)
*e*	6.94 (0.22)	5.34 (0.22)	5.32 (0.16)	5.36 (0.17)

In Section 3.2, the Metropolis algorithm was proposed as an alternative simulation method to estimate the variance of the LEs. We ran four sequences each of which contained 10000 simulated parameter vectors. For the starting values of the sequences we chose the MLE plus some random noise. The proposal density was multivariate normal and the algorithm was designed such that the acceptance rate was around 40%. Running the Metropolis algorithm for model 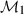 is computationally intensive given that there are 22 parameters to estimate.

We illustrate the results for the marginal and total LEs for men with mean education level. Fig. 2 shows the comparison with the MLE simulation for men aged 65, 75 and 85 years at baseline. The two methods produce similar results, which is reassuring with regard to the reliance on the asymptotic properties of maximum likelihood in the MLE simulation. For women the comparison (which is not reported) was similar. The MLE simulation estimated slightly wider densities (bigger variance) for *e*_2_, but numerically the differences were small.

To obtain the estimate for men aged 65 years there is a long period of extrapolation and small differences between methods will have an effect. As can already be inferred from the relatively low frequencies of observed transitions from state 1 to state 2 (Table 2), and from the relatively large estimated variance of the parameters for the transition intensity *q*_12_(*t*) (Table 3), estimation of *e*_12_ and *e*_2_ is subject to a relatively large variance. In general, the estimated LEs for the younger ages must be handled with care. Slight misspecification of the model can have a big effect on the estimation of the LEs because of the extent of the extrapolation.

As expected, men have a much lower overall LE than women at all ages. The amount of time that men spend with stroke is also lower at each age, though the total proportion of life spent with stroke is similar in men and women. Excluding individuals with cognitive impairment at baseline will have increased the LE of the remaining individuals as cognitive impairment is associated with poor survival.

### 5.3 Sensitivity analysis

The parameter estimates for models (7) and (8) rely heavily on model assumptions. This is often so in missing data analysis: values of *X*_*t*_ are only observed when *R*_*t*_=1, so estimates are driven by model assumptions rather than by evidence in the data (Little (1995), page 1115). To investigate the robustness of the missing data models, we discuss a sensitivity analysis that considers alternative link functions and functional form for the effect of age.

Models (7) and (8) are *logit* models where the link function is defined by logit(*p*)= log {*p*/(1−*p*)}. An alternative is the *probit* model that is defined by probit(*p*)=Φ^−1^(*p*), where Φ is the standard normal cumulative probability distribution. Another possibility is the complementary log–log-model defined by cloglog(*p*)= log {− log (1−*p*)}. By using alternative link functions and comparing results we can investigate—up to a certain extent—whether the models for the missing data are robust against misspecification of the link function.

Another sensitivity analysis is to investigate functional form for the effect of age in models (7) and (8), where the slope is assumed to be constant. The assumption can be tested by formulating a spline function that imposes piecewise constant slopes with possible slope change at so-called knots; see, for example, Greene (2002). For age in years, we choose knots *g*_1_,…,*g*_5_=71,77,83,89,95 and extend models (7) and (8) by adding covariates 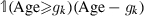 with regression effect *δ*_*k*_, for *k*=1,…,5. (In the implementation, we took the centring of age into account.) Another way to investigate functional form for the effect of age is to work with age categories. This can be used to investigate the assumption in models (7) and (8) that the effect of age is linear. The models were adapted by replacing the covariate age by five dummy variables for the categories 71–77, 77–83, 83–89, 89–95 and *older than* 95 years, and by using the category *younger than* 71 years as reference category.

The Akaike information criterion AIC can be used to investigate the alternatives formulated above. For model 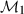 with the logit models we obtain AIC = 23997. Using the probit link we obtain AIC = 24001 and, using the cloglog-link, AIC = 23994. Using spline regression, AIC = 24026. Since model 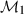 is nested in the model with the splines, the models can be compared by using the likelihood ratio test: the difference in minus two times the log-likelihood is 13.55, which is not significant (*p*-value 0.19). Using the dummy variables for age, AIC = 24021. The AICs for the models with different link models are similar, and the AICs for the spline regression and the regression with the dummy variables show that these extensions do not yield better models.

More relevant is to see how estimated LEs change across the different specifications. Because of the extrapolation, if there are differences, they will be most pronounced in the LEs of individuals aged 65 years old at baseline. Both for the probit model and for the cloglog-model, absolute differences for LEs for women and men are all smaller than 0.1 when compared with the results in Table 4. Also, for the spline regression and the regression with the dummy variables for age, absolute differences for LEs for women and men are smaller than 0.1.

The above summary shows that the estimation of the LEs is robust across some of the possible alternative specifications of the missing data model. Completely ignoring the missing data, however, does have a relevant effect on the estimation of LEs. This will be shown in the next section.

As stated at the end of Section 3.1, even though in some cases it is clear that the missing state is state 2, this state is not imputed before data are analysed. For the CFAS data at hand this is crucial. If we impute each of the 60 missing states of which we know that it must be state 2, selection model 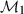 as formulated in the previous section is difficult to estimate: for submodel (8) point estimates for *γ*_2.0_ and *γ*_2.*R*_ are huge (positive and negative respectively) and the variance cannot be estimated as the Hessian is not positive definite. By using the restriction *γ*_2.*R*_=0, the model can be fitted, but estimated standard errors for submodel (8) are huge and hamper further statistical inference. This shows that, by not imputing these specific missing states, we maintain information about the missing data mechanism that is needed to estimate model 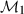.

### 5.4 Comparison with other models

In addition to the selection model 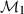, models 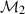 and 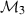 were introduced in Section 4. In what follows we compare the results for the three models with regard to the CFAS data. Although Section 4 showed that the models may yield different results, the true mechanism at work in the CFAS data is unknown as is how differences between parameter estimates affect estimation of LEs. We also consider the comparison between 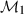 and Cox regression models (Cox, 1972).

Model 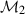 can be fitted to the CFAS data without any changes and data for model 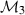 can be derived from the CFAS data by deleting all the intermittently missing states. Table 2 presents frequencies of observed transitions between the states in the CFAS when missing states are removed.

Parameter estimates for the models 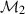 and 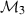 are presented in Table 3. If we assess the significance of the covariates by using univariate Wald tests, then we see that the three models differ in the estimation of the non-significant effects, especially for education. More education is associated with better survival and in that sense 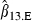 agrees with our expectation. But *β*_12.E_ and *β*_23_ are difficult to identify as is indicated by the relatively large standard errors. This may be due to the relatively small number of individuals who are observed in state 2. In our data analysis, there is no clear association between the risk of stroke and education. For the significant effects, point estimates are similar although they differ. The biggest difference is for the effect of age on the intensity of moving from state 1 to state 2 (*β*_12.A_). The increase of this effect follows the increase of the extent to which we model the missing states: 0.065 in model 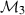, 0.078 in model 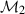 and 0.103 in model 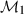. Since age increases for all individuals during follow-up, this means that modelling the missing states leads to a higher estimated risk of a transition from state 1 to state 2.

Reported estimated standard errors in Table 3 show a pattern: the precision of the estimation of covariate effects for the transitions from state 1 to state 2 and for the transitions from state 2 to state 3 is highest for model 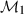. This concurs with the results in the simulation study where the means of the estimated standard errors (which are not reported in Table 1) showed that the selection model has a higher precision than the other two models with regard to the effect of age on transitions from state 1 to 2 and for state 2 to 3. By modelling possible non-ignorable missing states, the probability that a latent state is state 2 is larger (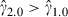) and hence more covariate information is used to estimate the effects for transitions to or out of state 2. Nevertheless, the differences in standard errors in Table 3 are small, and for some quantities models 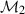 and 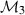 are more efficient than 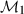; see, for example, intercept *β*_12.0_ and effect *β*_13.S_. Overall, combining the data analysis with the simulation study, the gain of modelling non-ignorable missing states is more about bias than precision.

For the comparison of the estimated LEs, see the results for the men in Table 4. If we use the estimated standard errors to construct 95% confidence intervals, then the intervals overlap across the models for all estimated LEs. This is reassuring regarding the effect of missing values. However, this does not conceal the fact that the models produce different figures. As was to be expected, estimates for those aged 65 years differ the most. This is due to the extrapolation—differences between the models become more apparent under extrapolation. The largest difference is for *e*_11_, where model 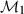 estimates 12.85 (0.37) and 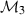 estimates 12.04 (0.25). Looking at the point estimates, model 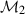 is an intermediate model, often producing estimates that are in between the estimates of 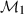 and 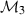. For the younger ages, model 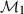 estimates higher LEs. For these ages, a missing state is less likely to be state 2 according to 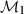 (Table 3: 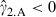). As a consequence, the possibility of a trajectory from an observed state 1 via a latent state 2 is less likely and mortality decreases as state 2 is associated with poorer survival.

In general, the comparison of a three-state model such as model 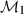 with a Cox regression model is of limited use. But here the idea is to fit two Cox models—one for each baseline state—and to compare survival estimated by the Cox models with survival estimated by the three-state model. This does not provide information on the risk of a stroke or on healthy LE, but it is a way to validate part of the three-state model: if there is not too much censoring, then estimated survival should be similar (see Siannis *et al.* (2007)). Of course, this is only a comparison between models and as such not a goodness-of-fit test. Given that observation times vary within and between individuals and that age is used as a time-dependent variable, we cannot apply Pearson-type goodness-of-fit tests as described in Aguirre-Hernández and Farewell (2002) and Titman and Sharples (2008).

Using the package in R, we fitted two Cox models with covariates age, sex and education as defined for model 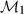. Age as a time-dependent variable was dealt with by rearranging the data such that every individually observed time interval is a record in the data. This is the *start–stop* format; see, for example, Venables and Ripley (2002), section 13.4. An event is defined as death and age within each record is age midway through the corresponding time interval.

**Fig. 3:**
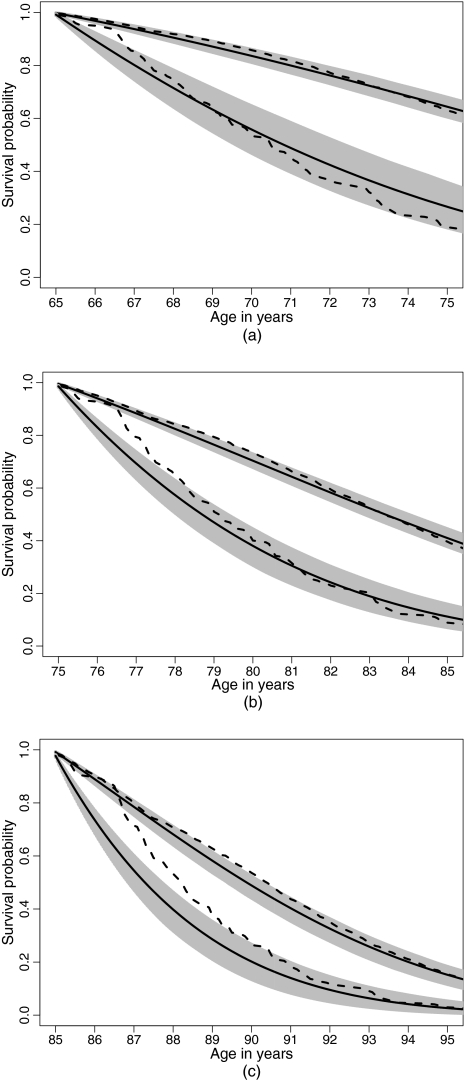
Fitted survival for model 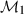 (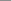; 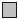, 95% confidence intervals) and for Cox regression models (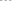) for men aged (a) 65 (b) 75 and (c) 85 years at baseline with mean education: in each graph the curves at the top are for baseline state 1 and the curves at the bottom are for baseline state 2

Fig. 3 depicts the results for men with mean education. For model 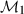, we used a piecewise constant approximation to estimate survival for an individual with covariates specified at baseline. This is the same approximation as used in the estimation of the LEs. Given time grid *u*_1_,*u*_2_,…,*u*_*M*_ and *h*=1 month, survival at time *u*_*j*_ for an individual in state *s* at baseline was estimated by 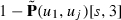, where 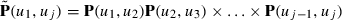. The 95% confidence intervals were derived from simulating model parameter uncertainty by using the MLE. For 500 simulated parameter vectors, survival was estimated and 95% confidence intervals were derived by using percentiles. For estimating survival given the Cox model, we used the same grid specification and used the function with the option (see Venables and Ripley (2002), section 13.4).

For men in state 1 at baseline, the two models estimate similar survival. The Cox model will always overestimate survival as it does not—for censored deaths—take into account a possible trajectory via state 2. For individuals in state 1 at baseline, 35% of the deaths are censored. (For individuals in state 2 at baseline, 11% of the deaths are censored.)

For men in state 2 at baseline, the two models estimate different survival for the older men —noticeably in the first half of the follow-up. This difference is partly due to left censoring. The Cox model analyses data from men who were in state 2 at baseline. Model 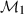 analyses in addition data from men who enter state 2 during follow-up. The former group will on average be less healthy than the latter group: hence the higher death rate in the first half of the follow-up according to the Cox model. For the men aged 65 years at baseline this problem does not occur. For this age group there are no men who enter state 2 during follow-up because 65 years is the minimum age at baseline. For women, the comparison between the models shows the same trend (which is not reported).

## 6 Conclusion

To take into account possible non-ignorable missing values, we fitted a selection model to the longitudinal data from the Medical Research Council CFAS. The selection model encompasses a continuous time three-state model to describe stroke history, and a missing data model to describe missing values. The model can be seen as an extension of the selection model in Cole *et al.* (2005). Our model allows for right censoring and, by modelling time continuously, exact death times are taken into account. The selection model can be used to estimate stroke-free LE. The goodness of fit was checked graphically by comparing model-based survival with the survival estimated by the semiparametric Cox regression model.

Whether or not missing states are taken into account has an effect on the estimates of LEs. Modelling the missing states as non-ignorable leads to higher estimated stroke-free LE for men aged 65 years and to lower estimated stroke-free LE for the men aged 85 years.

The analysis is restricted to individuals in Newcastle who are not severely cognitively impaired at baseline. This means that estimated LEs are a little higher than expectancies for the whole population in Newcastle. Reported total LEs are below the national UK average, but this is specific to Newcastle and in line with reported estimates elsewhere; see, for example, Matthews *et al.* (2006).

There are many ways in which informatively missing data can be ignored. The MAR model 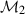 in this paper is rather specific—other MAR models are possible. The comparison between model 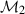 and the selection model 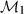 is not meant as a general comparison between MAR and missingness not at random models. A comparison which in general would not be very useful as every fit to observed data, obtained by using a missingness not at random model is exactly reproducible from an MAR model (Molenberghs and Kenward (2007), theorem 19.2). Model 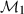 relies on model assumptions that cannot be linked to evidence in the data. For this reason, a sensitivity analysis was conducted to investigate functional form of the missing data model.

An issue that is not discussed in this paper even though it is quite common (and important) in longitudinal data analysis is missing values of covariates. There is no direct extension of our model to take into account missing covariate data. Since the model assumes covariates values to be fixed values, taking into account missing values of a covariate means introducing a distribution for the covariate—an exercise that is beyond the scope of this paper.

The model presented is flexible. It can be applied to data with more than three states and to data with backward transitions. The log-linear regression for the intensities makes it possible to model cohort effects by including calender year as a covariate, or to investigate risk factors that are associated with poor survival. The model is also intended as an alternative to the discrete time modelling that is implemented in the software IMaCh (Lièvre *et al.*, 2003). IMaCh has been used in research on healthy LEs (see, for example, Jagger *et al.* (2007) and Lièvre *et al.* (2008)), but the software always assumes that recovery from a disease or disability state is possible. In addition, modelling of non-ignorable missing values is not yet possible in IMaCH.
